# Quadriceps muscle strength recovery with the use of high tone power therapy after anterior cruciate ligament reconstruction: a randomized controlled trial

**DOI:** 10.1186/s12891-021-04862-w

**Published:** 2021-11-23

**Authors:** Katarzyna Ogrodzka-Ciechanowicz, Grzegorz Głąb, Jakub Ślusarski, Artur Gądek

**Affiliations:** 1 Institute of Clinical Rehabilitation, Faculty of Motor Rehabilitation, University of Physical Education in Krakow, Al. Jana Pawla II 78, 31-571 Krakow, Poland; 2 Trauma and Orthopaedics Clinical Department, University Hospital in Krakow, Krakow, Poland; 3 Department of Orthopaedics and Physiotherapy at Jagiellonian University Collegium Medicum, Krakow, Poland

**Keywords:** ACL injuries, Physical therapy modalities, Muscle strength dynamometer

## Abstract

**Background:**

There are no scientific reports clearly describing the effectiveness of the High Tone Power Therapy in patients after ACL reconstruction. This caused that in own research an attempt was made to present the possibilities of using the selected method of electrical stimulation in the treatment of an orthopaedic patient. The aim was to assess the effectiveness of electro stimulation of the quadriceps muscle in patients after ACL reconstruction, with the use of High Tone Power Therapy.

**Methods:**

In randomized controlled trial took part thirty-five men, aged 21–50, after ACL reconstruction. The tests were carried prior to and 6 months following the ACL reconstruction. After the surgery, the patients were randomly divided into experimental group (17 patients) with the High Tone Power Therapy in rehabilitation and control group (18 patients) without the High Tone Power Therapy. Patients were subjected to 6-month rehabilitation. Research tools included the measurement of muscle strength torque, ROM, knee and thigh circumference measurements, the Lysholm and the VAS scale.

**Results:**

After applying HiToP, the analysis showed a statistically significant improvement of muscle torque (*p* = 0.041, Es = 3.71), knee circumference (*p* = 0.039, Es = 1.65), thigh circumference (*p* = 0.049, Es = 1.26), knee extension (*p* < 0.001, Es = 2.20) in Experimental group compared to the control group. Only the results of the VAS scale did not differ statistically significantly both within a given group and between groups.

**Conclusions:**

The results confirm the hypothesis that the use of HiToP in patients after ACLr have a beneficial effect on muscle strength, reduction of joint effusion, muscle mass gain and joint function. The assumption that HiToP significantly reduces pain levels is not supported - the results in both groups are statistically insignificant.

**Trial registration:**

The research project was retrospectively registered in the Australian New Zealand Clinical Trials Registry (ANZCTR). Date of first registration 11.10.2016. Registration number: ACTRN12616001416482.

## Introduction

An injury to the anterior cruciate ligament (ACL) causes gradual weakness of the quadriceps strength by 5–40% [1]. The biggest weakness occurs in the first few months following the reconstruction and it most commonly affects the medial head of the quadriceps muscle. Restoration of muscle strength determines patient’s return to activity after reconstruction [1–3].

For many years neuromuscular electrical stimulation (NMES) has been applied in order to prevent muscular atrophy and restore their strength [4–7]. The primary effect of the NMES after ACL is restoration and improvement of the quadriceps function. This kind of therapy helps to activate the muscle whose activity is inhibited mostly by pain or effusion. In the literature there are a lot of contradictions regarding the effectiveness and benefits of using NMES in patients after ACL reconstruction (ACLr) [4–7].

NMES is used in the weakening of the quadriceps muscle after ACLr. Opinions differ regarding the time when NMES therapy should be included and how long it should be used to obtain a satisfactory effect – some researchers start NMES on the third day after surgery and continue until 4 weeks, and even up to 12 weeks after surgery [8–10].

However, the basic and main principle of using NMES is to restore and improve only the function of the quadriceps. Research is being done to prevent weakness in the quadriceps or the effects of NMES on the regeneration of the quadriceps muscle, or to re-educate the muscle or delay its atrophy. Others described the desired effect of the treatment as a contraction of sufficient intensity to re-educate the quadriceps [5, 11, 12].

NMES focuses only on the effects of electrostimulation on muscle function. The relatively new method of electrotherapy is High Tone Power Therapy (HiToP) has a much wider application. Its undoubted advantage is the ability to influence the whole body. It directly affects cell metabolism, improving metabolic processes in tissues, and pain relief. It can also be used to obtain stimulating effects of the current, in this case also for electro stimulation of the quadriceps [8–10].

NMES stimulates the nerves and muscles [13]. While, the main goal of HiToP is a direct effect on the cell’s metabolism. The differences between the two therapies are visible in three aspects: effect, technology and practical application. The differences in effect relate to the two main mechanisms of HiToP: First, HiToP introduces energy into the body to increase cellular energy potential. Secondly, it causes oscillations in cell structures that normalize metabolism. From a technical point of view, NMES is only modulation of amplitude, i.e. the current intensity is modulated but the frequency remains constant. Electrotherapy uses modulation frequencies from 0 to 200 Hz in the low frequency range and mainly 4000 Hz in the mid-range frequency range. In HiToP, both amplitude and frequency are modulated simultaneously. The higher the frequency, the more energy can be introduced corresponding to the individual threshold curve of the patient’s electrosensitivity. Differences in practical application:

1. NMES is applied for 5 to 10 min, and HiToP for up to 60 min.
2. In NMES, as a rule, one channel and two electrodes are used. In HiToP up to 4 channels are in parallel operation, with at least 10 electrodes [13].

Up to date, to the best of our knowledge, there are no scientific reports clearly describing the effectiveness of the HiToP therapy in patients after ACLr. This caused that in own research an attempt was made to present the possibilities of using the selected method of electrical stimulation in the treatment of an orthopaedic patient.

The aim of the research was to assess the effectiveness of the quadriceps muscle electro stimulation in patients after ACLr, with the use of HiToP therapy and above all answering the following questions:

1. What impact on the analyzed variables in patients after ACLr had the use of HiToP compared to the Control group?
2. How were pain and functional level assessed in both groups?

Based on the above questions, the following hypotheses were adopted:

1. The use of HiToP in patients after ACLr will have a beneficial effect on the selected variables in Experimental group.
2. Pain and functional level of patients enrolled in the study, who received HiToP, will improve compared to the Control group.

## Methods

### Design

This randomized controlled trial was reported according to the recommendations of the Consolidated Standards of Reporting Trials (CONSORT) statement [14].

Measurements were carried out between 2017 and 2019 at the University of Physical Education in Laboratory of the Diagnostics of the Motor System, Laboratory of the Motion Analysis, in collaboration with the Trauma and Orthopaedics Clinical Department, University Hospital in Krakow.

The first author enrolled patients from a list of patients for ACLr. Patients qualified for the research were patients of the Trauma and Orthopaedics Clinical Department, University Hospital in Krakow.

Each patient was randomly assigned to one of two groups on the day the ACL reconstruction surgery was completed. Before starting the intervention, patients were randomly allocated to the Experimental or Control group by an independent researcher using the sealed envelopes method. The first group had the HiToP included in rehabilitation (Experimental group), while the second group, had been treated without the HiToP (Control group).

Eligibility criteria for the research were: isolated, complete ACL rupture confirmed by imaging examination (Magnetic Resonance imaging), the form of surgery prescribed by a physician – ACLr by an autogenic method – ST tendon graft, ability to move independently before and after the surgery (without the use of orthopaedic supports), no other injuries or illnesses that may affect the outcome of the measurements e.g. damage to the menisci, degenerative changes in the joints (tests performed by an orthopedist). The patients had not taken medications affecting motor coordination and after becoming acquainted with the purposes and the course of the research – a voluntary consent to participate in the study (written consent). Patients also declared that they would not participate in any other physiotherapy.

Criteria for exemption from the surgery and rehabilitation program were: more than two absence in the rehabilitation program and interruption of continuity of the graft.

Patients informed and written consent was obtained, and the rights of subjects were protected. The study was conducted according to the guidelines of the Declaration of Helsinki, and approved by the Ethical Committee of Regional Medical Chamber in Krakow (No. 19/KBL/OIL/2014).

### Intervention

In the period from 1 to 10 day after the surgery the patients complied with the physician’s recommendations: the knee was blocked in the orthosis in full extension (0°), cooled the joint with ice cubes (twice a day for 15 min.), weight-bearing as tolerated and quadriceps contraction. Post-operative restrictions were put in place to provide protection to the repaired ligament.

Next patients from both groups started intensive physiotherapy. Both groups had an identical exercise program. During the first 3 months physiotherapy was carried out 3 times a week, while between 4th and 6th month – twice a week. Physiotherapy included general rehabilitation and exercises focused on the knee function. The exercise protocol included exercises improving the ROM, strengthening the muscles of the operated limb, proprioception and coordination exercises with the use of accessories, i.e. TheraBand strip. Strengthening exercises were selected individually – the load increased gradually with an increase in the range of motion and muscle control (from no load to at least 90% of the healthy leg). The number of repetitions was 10–15, patients started with 2–3 series, ending in the last month with 6 series. The therapy started with isometric exercises and SLR (straight leg raise) and ended with running, split squat jumps and single leg drop landing. (Table 1.)

**Table 1:**
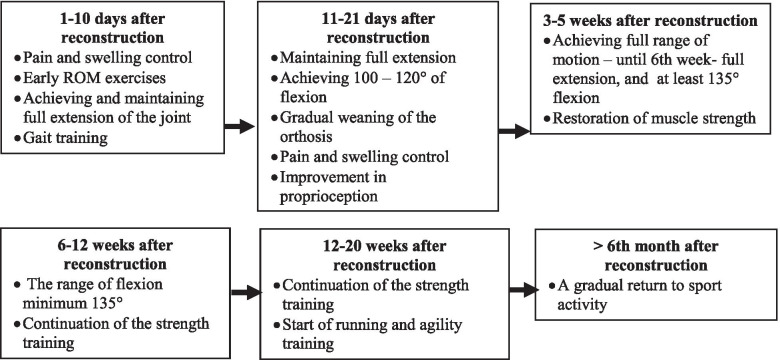
The goals of the individual stages of physiotherapy

Patients from Experimental group were also applied HiToP® (Ekomedica, Poland). HiToP treatment involved electro stimulation of the quadriceps muscle at a frequency of 20 Hz (two electrodes set in the place of transition of the belly of the muscle into a tendon) and vitalisation: electrodes were arranged on the feet, forearms and the cervical spine using simultaneous frequency and amplitude sweep (in the range from 4096 to 32,768 Hz) which purpose was to trigger and use in the therapy non stimulating effects of the currents.

The therapy is based on bi-directional currents of average frequency (4096–32,768 Hz). Such currents cause stimulating effects (they trigger off action potential for example in muscles, nerves or receptors) and unstimulating effects, namely influence on metabolism. Unlike other methods employed in electrotherapy, in the HiToP therapy there is a simultaneous change in amplitude and frequency. This allows to get unstimulating effects by applying high doses. In spite of this, the therapy alone does not cause bigger sensations in the patient. What is more, side effects in the form of irritation or burning are reduced [15–17].

There are two modes of operation in the HiToP:

- “Simul FAMi”
- “Simul FAM X”.

“Simul FAMi” is based on non-stimulant effects, that is a high-frequency current which causes the formation of electric field in tissues which later vibrates different molecules, for example, dipoles or ions. It works in the tissues as a biocatalyst improving the diffusion of ions and affecting the distribution of substances such as mediators of pain or products of metabolism, which results in the reduction of pain. In addition to analgesia, medium frequency currents cause the acceleration of chemical reactions and metabolism which stimulates tissue regeneration. Researchers also highlight the influence of the medium frequency currents on resorption of hematoma and effusions by increasing circulation (both venous and lymphatic) and greater permeability of cell membranes [15–17].

The “Simul FAM X” mode, depending on the frequency, allows the use of stimulant effects (e.g. nerve or muscle stimulation). It involves triggering an action potential (nerves, muscles, or receptors). The range of stimulant frequencies which are most commonly used in the HiToP is from 3 Hz to 100 Hz [15–17].

Both stimulant and non-stimulant effects are used in therapy. Non-stimulant effects are mainly used in conditions where there is a need to accelerate tissue regeneration or improve metabolic processes, ie. in chronic diseases such as rheumatism or arthritis or in overload or after soft tissue injuries [15–18]..

Local or global application can be used in the HiToP. The device is usually equipped with 4 channels running independently of each other. The duration of treatment varies from 20 min to 60. It is recommended to perform 10 treatments daily or every other day [15].

HiToP was performed after each physiotherapy sessions and the duration of administration was 1 h. 1 h of HiToP session took place after the completion of 1 h of physiotherapy. The total duration of intervention in patients from the Experimental Group was 2 h.

The therapist responsible for the physical therapy sessions was blind, i.e. he knew the physiotherapy program, but did not know the scientific assumptions of the project.

### Outcome measures

The study included measurements of maximum muscle torque of the quadriceps and knee extension range of motion (ROM), evaluation of knee function and pain assessment.

Each group was measured twice. The first examination session took place 2 days prior to the surgery.

Muscle strength and ROM measurements, assessment of pain and knee function were repeated after 6 months. The second examination session took place 2 days after the end of physiotherapy.

### Primary outcome

**Fig. 1:**
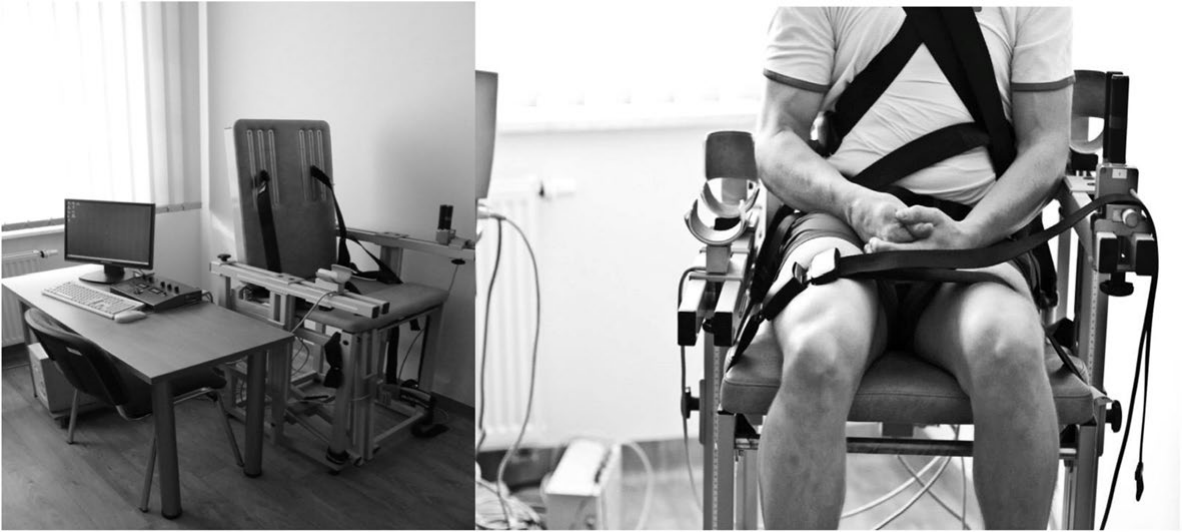
Station for the moments of maximum muscle strength test [own source]

For each patient measurements of the maximum muscle torque were performed in standard sitting positions for the measurement of the knee extensors strength (angles between the trunk, hip joint and knee joint were 90°). Stabilization with a belt was placed on the shoulders, chest, pelvis and thighs. During the examination, the upper limbs were crossed over the chest. The setting of the chair and the dynamometer with the attachment complied with the standards specified by the manufacturer and was adjusted individually for each test subject (Fig. 1). The dynamometer axis was set at the level of the knee joint space (the joint rotation axis). During the measurement, the patient was motivated verbally as well as visually by observing the current torque graph on the screen. According to the manufacturer, the possible measurement error does not exceed 0.02%. The best result obtained by the examined person was taken into account for the statistical analysis. The duration of recording one measurement is 10 s. Each patient had 2 tests. There was a 10-min rest break between the measurements.

The person conducting the research did not know the patients and was not involved in their treatment.

The examination was conducted by means of the isometric muscle force measurement and analysis program, 2001 Metitur Ltd. The value of maximum muscle force (Fmax) was measured and on its basis the values of maximum muscle torque (τmax) and relative muscle torque (τr) for a selected group of muscles were calculated according to:

$$\uptau \mathrm{max}=\mathrm{Fmax}\ \mathrm{x}\ \mathrm{d}\ \left[\mathrm{Nm}\right]$$

$$\uptau \mathrm{r}=\uptau \mathrm{max}/\mathrm{m}\ \left[\mathrm{Nm}/\mathrm{kg}\right]$$

where: d-the value of the external force arm- the distance from the axis of joint rotation to the line of action of the dynamometer, m-weight of the subject [19].

### Secondary outcome

measurements of the knee extension range of motion were carried out using a goniometer, measurement of the circumferences of the thigh (10 cm from the base of the patella) and the knee and Lysholm scale were performed, as well as the visual-analog pain assessment scale (VAS) [20–22].

The Lysholm Scale consists of eight items that measure: pain (25 points), instability (25 points), locking (15 points), swelling (10 points), limp (5 points), stair climbing (10 points), squatting (5 points), and need for support (5 points). Every question response has been assigned an arbitrary score on an increasing scale. The total score is the sum of each response to the eight questions, and may range from 0 to 100. Higher scores indicate a better outcome with fewer symptoms or disability. Then an assignment is given as “excellent” for 95 to 100 points; “good” for 84 to 94 points, “fair” for 65 to 83 points, or “poor” for less than 65 points [21].

### Data analysis

Methods of descriptive statistics were used to present the results in the form of tables containing arithmetic means, standard deviation, minimum and maximum values.

The STATISTICA 12.0.PL software was used for statistical analysis. The first stage of the analysis was to check the normality of the distribution of variables using the Shapiro-Wilk test. The next step was to determine the significance of changes between the variables. Two-way ANOVA analysis (ANOVA group x time) was used. Multiple comparisons were based on the Bonferroni correction. Cohen’s d allowed for the assessment of the effect size, which was analyzed in accordance with previous studies [23–25]. Cohen’s d was defined as the difference between two means divided by a standard deviation for the data:

$$d=\frac{{\overline{x}}_1-{\overline{x}}_2}{s}$$

were *d* = 0.2 is considered a ‘small’ effect size, 0.5 represents a ‘medium’ effect size and 0.8 a ‘large’ effect size [24, 25]*.*

The study assumed *p* < 0.05. The paired t-test power analysis showed that at least 15 subjects (in each group) were needed to obtain a power of 0.8 at a two-sided level of 0.05 at an effect size of d = 0.6.

## Results

**Fig. 2:**
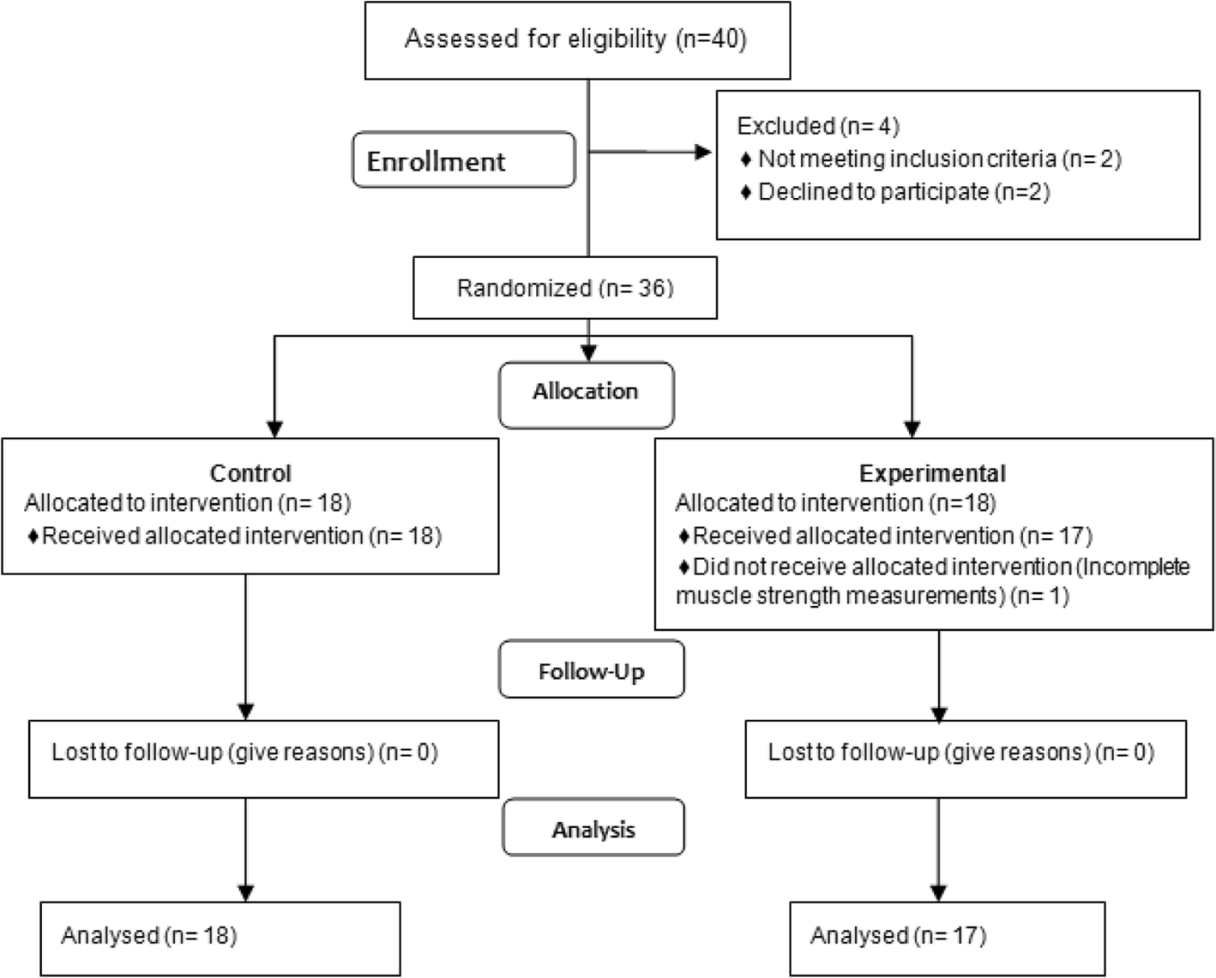
Flow diagram

40 patients were recruited but 36 were eligible to be included in the study. In Control group 18 patients were followed up to analysis and in Experimental group 18 participants were allocated to intervention but one of them lost to follow-up up (95% follow-up). Finally research material comprised a group of 35 men, aged 21–50 (mean age 28.4 ± 7.83). Characteristics of patients divided into two groups are displayed in Table 2. Figure 2. presents the qualification process for research.

**Table 2:** Anthropometric data

Variable	Experimental			Control			***p***
	X	SD	95% CI	X	SD	95% CI	
**Age [yrs]**	30	7.35	26.5–33.6	30	10.42	25.2–34.8	0.88
**Body height [cm]**	175.74	3.52	174–177	177.74	8.01	174–181	0.41
**BMI**	25.53	3.47	23.9–27.2	24.42	3.54	22.8–26.1	0.43

Experimental – patients with HiToP in physiotherapy; Control – patients without HiToP in physiotherapy

The conducted analysis showed a statistically significant increase in the measurement of the muscle torque in the experimental group (*p* = 0.041, Es = 3.71). The Control group did not change significantly, and even the muscle torque measurement was weaker after physiotherapy. The difference between the results of the II measurement between the groups was also statistically significant in favor of the Experimental group (*p* = 0.028, Es = 4.47).

The result of the measurement of the knee circumference showed a significant increase in the Experimental group (*p* = 0.039, Es = 1.65). The result in the Control group was statistically insignificant. The analysis also showed a significant difference between the groups in the II measurement, in favor of the Experimental group (*p* = 0.043, Es = 1.50).

The results of the thigh circumference measurement also show a significant increase after the application of rehabilitation with HiTop in the Experimental group (*p* = 0.049, Es = 1.26). The result in the Control group before and after rehabilitation was statistically insignificant. The difference between the results of both groups in the II measurement was also statistically significant in favor of the Experimental group (*p* = 0.033, Es = 1.42).

The analysis showed a significant increase in the knee extension in the Experimental group (*p* < 0.001, Es = 2.20). In the Control group, the result was statistically insignificant. The comparison of the results of the II measurement shows a statistically significant difference between the groups, in favor of the Experimental group (*p* = 0.048, Es = 4.23). (Table 3.)

**Table 3:** Comparison of outcome variables characterizing the knee indicators at baseline and after physiotherapy

		Baseline	95% CI^**a**^	Post	95% CI^**a**^	***p***^***a***^	ES^**a**^
		X ± SD		X ± SD			
**Knee extensors strength** **[N/kg]**	**Experimental**	20.49 ± 0.8	20.1–20.9	23.28 ± 0.7	23–23.7	**0.041**	3.71
	**Control**	20.30 ± 0.6	20–20.6	20.19 ± 0.6	19.9–20.5	n.s.	0.18
	***p***^***b***^	n.s.		**0.028**			
	**ES**^**b**^	0.26		4.47			
	**95% CI**^**b**^	−0.29-0.67		2.64–3.53			
**Knee circumference [cm]**	**Experimental**	39.00 ± 2.90	37.6–40.4	35.00 ± 1.80	34.1–35.9	**0.039**	1.65
	**Control**	39.00 ± 1.84	38.1–39.9	38.00 ± 2.18	37–39	n.s.	0.50
	***p***^***b***^	n.s.		**0.043**			
	**ES**^**b**^	0.65		1.50			
	**95% CI**^**b**^	−1.66-1.66		1.62–4.38			
**Thigh circumference [cm]**	**Experimental**	41.00 ± 4.02	39.1–42.9	46.00 ± 3.86	44.2–47.8	**0.049**	1.26
	**Control**	42.00 ± 3.80	40.2–43.8	41.00 ± 3.11	39.6–42.4	n.s.	0.28
	***p***^***b***^	n.s.		**0.033**			
	**ES**^**b**^	0.25		1.42			
	**95% CI**^**b**^	−1.69-3.69		2.6–7.4			
**Knee extension [°]**	**Experimental**	5.00 ± 3.20	3.4–6.5	0.00 ± 0.15	−0.07-0.07	**< 0.001**	2.20
	**Control**	4.00 ± 2.65	2.7–5.2	3.00 ± 0.99	2.54–3.46	n.s	0.49
	***p***^***b***^	n.s.		**0.048**			
	**ES**^**b**^	0.34		4.23			
	**95% CI**^**b**^	−1.02-3.02		2.51–3.49			

Baseline – the measurement before physiotherapy; Post - the measurement after physiotherapy; *p*^*a*^ *– p-*value between baseline and post-physiotherapy within each group*; p*^b^ – *p*-value between study groups*;* ES^a^ – effect size (Cohen d) within each group; ES^b^ – effect size (Cohen d) between study groups*; p* < 0,05; *CI*^a^ Confidence interval within each group based on means and SD*, CI*^b^ Confidence interval between study groups based on means and SD.

The analysis showed that within the groups the Lysholm scale results increased after physiotherapy and these differences were statistically significant. The difference between the groups in the II measurement was statistically significant in favor of the Experimental group *p* = 0.035, Es = 1.13).

Comparison of results of pain level according to the VAS scale in I and II measurement in the Experimental group and the Control group showed no statistically significant differences. The results of the pain scale in both I and II measurement showed that the patients from the Experimental group determined the degree of pain according to the VAS scale at a lower level than the patients in the Control group. The differences, however, were statistically insignificant. (Table 4.)

**Table 4:** Statistical analysis of the results of the Lysholm and VAS scales in both Groups at baseline and after physiotherapy

		Baseline	95% CI^**a**^	Post	95% CI^**a**^	***p***^***a***^	ES^**a**^
		X ± SD		X ± SD			
**Lysholm scale [pts]**	**Experimental**	65 ± 4.20	63–67	94 ± 7.01	90.7–97.3	**0.011**	5.01
	**Control**	66 ± 6.33	63.1–68.9	85 ± 8.71	81–89	**0.021**	2.49
	***p***^***b***^	n.s		**0.035**			
	**ES**^**b**^	0.18		1.13			
	**95% CI**^**b**^	−2.72-4.72		3.54–14.46			
**VAS scale [pts]**	**Experimental**	1 ± 0.5	0.7–1.2	2 ± 0.4	1.8–2.2	n.s.	2.20
	**Control**	2 ± 0.2	1.9–2.1	3 ± 0.8	2.6–3.4	n.s.	1.71
	***p***^***b***^	n.s		n.s			
	**ES**^**b**^	2.62		1.58			
	**95% CI**^**b**^	0.76–1.26		0.56–1.44			

Baseline – the measurement before physiotherapy; Post - the measurement after physiotherapy; *p*^*a*^ *– p-*value between baseline and post-physiotherapy within each group*; p*^b^ – *p*-value between study groups*;* ES^a^ – effect size (Cohen d) within each group; ES^b^ – effect size (Cohen d) between study groups*; p* < 0,05; *CI*^a^ Confidence interval within each group based on means and SD*, CI*^b^ Confidence interval between study groups based on means and SD.

## Discussion

The aim of the research was to assess the effectiveness of the quadriceps muscle electro stimulation in patients after ACLr, with the use of HiToP therapy. The obtained results indicate the effectiveness of the applied HiToP therapy. The quadriceps muscle torque has improved, the circumference of the knee has decreased, which may mean a reduction in joint effusion, while the thigh circumference has increased. It should be noted, however, that measuring the quadriceps circumference using tape is a result that suggests changes in muscle mass but not unambiguously determining the increase in muscle mass. The results of the Lysholm scale indicate a significant improvement in the function of the knee joint in both groups, however, the result in the Experimental group, where HiToP was applied, was higher, i.e. the improvement was more pronounced than in the Control group. There was no significant improvement in the assessment of the pain scale, measured by the VAS scale. However, in both groups, the pain assessment in both measurements (Baseline and Post) was low, i.e. pain was not significantly felt. These results clearly indicate that the inclusion of HiToP in the physiotherapy program after ACLr brings significant benefits in returning to the patient’s fitness.

Injury of the ACL mostly concern young and active people who get injured during recreational or professional physical activity. Comprehensive physiotherapy covering both kinesitherapy and physical therapy is aimed at restoring motor skills in patients after ACLr. The mobility depends on the adequate level of knee muscles strength, range of motion, proprioception. Immediately after the reconstruction the range of motion in the knee joint is limited by pain and swelling. Such a limitation does not have a positive influence on muscle strength, particularly knee extensors. Researchers agree that after rehabilitation the values of muscle strength should be similar to those from before the surgery [26–29].

One of the elements of therapy of patients after ACLr can be electrostimulation of the quadriceps muscle, which helps to rebuild muscle strength. Currently, in the rehabilitation of patients after ACLr HiToP is used, which in addition to muscle stimulation supports cell metabolism, thereby reducing pain [15].

In the available literature there is, however, lack of reports on the effectiveness of the use of the HiToP in patients after ACLr. There are also no publications including the analysis of the results of knee extensors strength measurements in patients after ACLr in whom the HiToP was used. Which makes our own research a significant contribution to the assessment of the effectiveness of HiToP in the treatment of patients with motor organ injuries.

One of the few authors who undertook the evaluation of the effectiveness of HiToP in rehabilitation of patients with soft tissue injuries was Wilk et al. They indicate the improvement in muscle strength of knee extensors after including the HiToP therapy into rehabilitation [9]. The results of our own research may confirm this, as the patients from the Experimental group obtained significantly better results in quadriceps muscle torque. It is related to the stimulation effect of HiToP (Simul FAM X).

Also in the studies of Janiszewski et al., who analyzed the use of HiToP in patients with pain in the lumbar spine, after using the therapy, the strength of the muscles stabilizing the spine improved as well as lumbar mobility [29]. Kulis et al. also confirm the effectiveness of HiToP in the therapy of patients with cervical spine pain [30]. The authors conducted a study on a group of 40 people who used HiToP twice a week for 30 min for a period of 3 weeks. Test results show an improvement in the mobility of the cervical spine and a reduction in back pain. The impact of the HiToP on the range of motion was analyzed by Wilk et al. who, after the therapy, found improvement in the knee range of motion [16].

The results of our research refer to patients after ACLr, but also here an improvement in the knee extension was achieved, which was related, i.a., to an improvement in the quadriceps strength and a decrease in the knee circumference, which indirectly indicates the anti-edema effect of HiToP. This effect results directly from the non-stimulating effect of HiToP (Simul FAMi), which improves blood circulation and increases cellular metabolism. This is a significant advantage of HiToP over NMES. Nowakowska et al. in their study observed increased blood flow in the microcirculation of the lower limbs after applying the HiToP [17]. Which also confirms the results of own research.

One of the issues that were addressed in this study was the influence of the HiToP on pain. Wilk et al., on the grounds of their studies, conclude that pain is reduced in patients after the HiToP [16]. Analysis of subjective evaluation of pain in patients after ACLr showed reduction of pain in both tested Groups. Better results were obtained by patients whose rehabilitation included the HiToP therapy, but the differences between measurements and between Groups were statistically insignificant.

Comparing the results of this research with the results of studies on electrostimulation of the quadriceps, it is worth noting that HiToP has a wider application (stimulating and unstimulating effects). This causes in the HiToP therapy there is a simultaneous change in amplitude and frequency. This solution allows you to get unstimulating effects by applying high doses. In spite of this, the therapy alone does not cause bigger sensations in the patient. This is one of the differences that distinguishes HiToP from other electrostimulations [15].

Hauger et al. Suggest that traditional NMES has a positive effect on the restoration of the strength of the quadriceps muscle after ACLr in the early postoperative period. The results of this research indicate, in turn, that HiToP has a long-term effect – the study was performed after 6 months of therapy and allowed to conclude that the strength of the muscle has significantly improved [12].

In addition, it is suggested that NMES also has a short-term effect on joint function assessed, for example, by the Lysholm scale. Most studies show that this effect does not exceed 6 weeks of observation [7, 31, 32]. In this reserch, the results clearly indicate the long-term effect of HiToP on the joint function, which was tested with the Lysholm scale.

The results of this research indicate that the effect of HiToP in terms of both electrostimulation and cell metabolism causes long-term effects on the quadriceps and knee joint function after ACLr. Thanks to the assessment after 6 months from ACLr and long-term observation of patients who received HiToP, improvement in muscle strength can be noticed compared to standard physical therapy alone.

### Study limitations

Analysis of studies of other authors shows the positive impact of the HiToP on the strength and function of the knee in patients after ACLr. This study is not without limitations. An important aspect that could affect trial results is determining the level of the patients’ physical activity before injury and extend the research also to the group of women.

Also measuring the circumference with the use of the tape is an indirect method and cannot be the only way to measure the increase in muscle mass. However, it is a guideline on the basis of which it is worth extending the research, for example by applying an imaging exam.

There are few scientific reports which would show that the inclusion of the HiToP therapy into the rehabilitation program gives better results. The obtained results of own research (especially in such a long-term physiotherapy – 6 months) clearly indicate that from the therapeutic point of view is an important signal that the use of HiToP therapy in rehabilitation of patients after ACLr as well as after other surgeries or injuries brings positive results. Therefore, it is worth to expand this issue in future.

It is also worth comparing the HiToP results with another type of electrostimulation, which could clearly define the effect of HiToP on the improvement of the function of the quadriceps muscle. This topic will be considered in the authors’ further research.

## Conclusions

The research results confirm the hypothesis that the use of HiToP in patients after ACLr have a beneficial effect on muscle strength, reduction of joint effusion, muscle mass gain and joint function. The assumption that HiToP significantly reduces pain levels is not supported - the results in both groups are statistically insignificant.

### Practical implications

The use of HiToP in the treatment of patients after ACLr allows for faster regeneration, improvement of the knee joint function, and thus should become a standard in the rehabilitation process of patients after ACLr.

## Data Availability

The datasets used and/or analysed during the current study are available from the corresponding author on reasonable request.

## Abbreviations

ACL: Anterior Cruciate Lgament
ACLr: Anterior Cruciate Ligament reconstruction
HiToP: High Tone Power therapy
NMES: Neuromuscular electrical stimulation
ROM: Range of motion.
